# Preoperative circulating tumor cells to predict microvascular invasion and dynamical detection indicate the prognosis of hepatocellular carcinoma

**DOI:** 10.1186/s12885-020-07488-8

**Published:** 2020-10-31

**Authors:** Jiangmin Zhou, Zhiwei Zhang, Honghao Zhou, Chao Leng, Bingwu Hou, Chenyang Zhou, Xinsheng Hu, Jinlin Wang, Xiaoping Chen

**Affiliations:** 1 Hepatic Surgery Center, Tongji Hospital, Tongji Medical College, Huazhong University of Science and Technology, 1095 Jiefang Avenue, Wuhan, 430030 China; 2 Translational Medicine Center, Tongji Hospital, Tongji Medical College, Huazhong University of Science and Technology, Wuhan, 430030 China

**Keywords:** Circulating tumor cells, Microvascular invasion, Isolation by size of epithelial tumor cells, Hepatocellular carcinoma, Hepatectomy, Extrahepatic metastasis

## Abstract

**Background:**

This study explored the diagnostic power of preoperative circulating tumor cells (CTCs) for the presence of microvascular invasion (MVI) and the relationship between dynamic changes in postoperative CTCs and prognosis.

**Methods:**

A total of 137 patients were recruited for the study. Preoperative blood samples were collected from all patients to detect CTCs. The time points for blood collection were before the operation, during the operation, and at 1 week, 1 month, 2 months, 3 months, 6 months, and 1 year after surgery. The predictive power of CTC count for the presence of MVI was analyzed by receiver operating characteristic (ROC) curve analysis. According to recurrence status, 137 patients were divided into three groups: no recurrence, early recurrence, and non-early recurrence groups.

**Results:**

A threshold CTC count of 5 showed the most significant power for predicting the existence of MVI. In multivariate analysis, the parameters of preoperative CTC count, alpha-fetoprotein (AFP) and tumor diameter were independent predictors of MVI (*P* <  0.05). A CTC count greater than or equal to 5 had better predictive value than AFP > 400 μg/L and tumor diameter > 5 cm. The number of intraoperative CTCs in the three groups did not increase compared to that before surgery (*P* > 0.05). The number of CTCs in the nonrecurrence group and the non-early recurrence group decreased significantly 1 week after surgery compared with the intraoperative values (*P* <  0.001), although there was no significant difference in the early recurrence group (*P* = 0.95). Patients with mean CTC count ≥5 had significantly worse long-term outcomes than those with mean CTC count < 5 (*P* <  0.001).

**Conclusion:**

The preoperative CTC counts in the peripheral blood of patients with HCC are closely correlated with MVI. The intraoperative manipulation of the lesion by the surgeon does not increase the number of CTCs in peripheral blood. Surgical removal of the tumor decreases the number of CTCs. The persistence of CTCs at a high level (≥ 5) after surgery suggests a risk of early recurrence.

**Clinical trial registration:**

Registration number is ChiCTR-OOC-16010183, date of registration is 2016-12-18.

## Background

Hepatocellular carcinoma (HCC) is one of the most common malignancies, and its high mortality makes it the second leading cause of cancer death worldwide [1]. The dismal prognosis of HCC has improved significantly over the last decade due to the increased knowledge of its behavior, improvements in staging systems and multiple therapeutic options [2]. Nevertheless, the prognosis of HCC remains very poor due to the high incidence of recurrence and metastasis; the 5-year recurrence rate after curative treatment remains high (70%), with 15% of patients with HCC developing extrahepatic metastases [3]. One important reason is that tumor cells are able to penetrate the microvasculature, disseminate through the bloodstream to other sites and finally form metastatic tumors. Studies have suggested that microvascular invasion (MVI) in HCC is one of the most significant risk factors for recurrence and metastasis following curative surgical resection [4]. MVI is defined as clusters of cancer cells observed microscopically in vessels located in the tumor capsule and surrounding liver parenchyma [5]. When a major lesion is removed, residual micrometastases could form postoperatively and result in early recurrence. Recent studies have demonstrated that tumor diameter, tumor number, degree of differentiation and serum level of prothrombin induced by vitamin K absence-II (PIVKA-II) were predictors of MVI [6]. One study reported that independent predictors of MVI included tumor diameter > 2 cm, alpha-fetoprotein (AFP) > 200 ng/mL and PIVKA-II > 40 mAU/mL [7]. Previous research has reported that the incidence of MVI ranged from 15 to 57% in HCC specimens and was associated with tumor size, AFP and typical imaging features [8]. The presence of MVI increases the risk of recurrence and dramatically shortens long-term survival [9]. Although preoperative imaging can evaluate macrovascular invasion and is helpful for selecting the appropriate therapy, MVI is almost impossible to predict by traditional preoperative imaging and is confirmed only by histopathological diagnosis after surgery. Therefore, the application of predictors of MVI is still very limited when selecting therapeutic strategies and predicting prognosis [10].

Circulating tumor cells (CTCs) are cancer cells that circulate in the bloodstream after being shed from the original tumor or metastatic foci, leading to new metastasis [11]. By virtue of the properties of the epithelial-to-mesenchymal transition (EMT) process, CTCs eventually evolve into a proportion of mesenchymal HCC cells, which are more metastatic than epithelial cells [12, 13]. In addition, several CTCs aggregate to form CTC clusters, which have a significantly greater potential to play an important role in cancer metastasis than single CTCs [14]. The liquid biopsy technique represented by CTC detection has great potential to contribute to the implementation of precision medicine in patients with HCC. CTCs obtained by the liquid biopsy technique can be used to evaluate the presence of MVI. A meta-analysis showed that CTC positivity was significantly associated with vascular invasion [risk ration (RR) 1.99, 95% CI: 1.43–2.77; *P* <  0.001)] [15].

In addition, resection and liver transplantation are still the mainstream methods for the treatment of HCC. Some studies have shown that tumor cells tend to disseminate from the vascular portal and are driven into the bloodstream when moving and rotating the liver [16]. Previous studies showed that an increase in postoperative CTC counts was associated with liver resection [17]. An increased number of CTCs after surgery was an independent predictor of recurrence [18]. In contrast, some studies observed a significant decrease in CTCs after the primary tumor was resected when the values were compared to those taken during the preoperative stage [19]. Therefore, the correlation of postoperative CTC counts with surgical procedures remains controversial.

At present, there are few studies on preoperative CTC counts as a predictive indicator of the presence of MVI, and there are also few studies on the changes in postoperative CTC number. In this paper, the optimal cut-off value of CTCs for predicting MVI was evaluated through preoperative CTC counts, and the change in the number of CTCs after surgery explained how postoperative recurrence and death were affected. This information will contribute to understanding the impact of changes in the number of CTCs on prognosis. In the future, CTCs may be used as a clinical indicator to monitor recurrence and death.

## Methods

### Trial design

This study was an observational clinical study. The patients were divided into MVI-positive and MVI-negative groups to evaluate the diagnostic power of parameters for the presence of MVI. We divided the patients according to the recurrence interval with regard to the relationship between postoperative CTC count and recurrence. The same inclusion and exclusion criteria, surgical criteria, and CTC testing criteria were followed throughout the study. No criteria were changed during the study.

### Patients

Patients were recruited from the Hepatic Surgery Center, Tongji Hospital, Tongji Medical College, Huazhong University of Science and Technology. The inclusion criteria were as follows: 1) definitive pathological diagnosis of HCC based on the World Health Organization criteria; 2) curative resection, defined as the complete macroscopic removal of the tumor with negative margins (R0) [20]; 3) no prior anticancer treatment; and 4) age between 18 and 80 years. The exclusion criteria were as follows: 1) distant metastasis; and 2) Child-Pugh C liver disease. In addition, 18 patients with benign liver disease were enrolled as negative controls. All procedures performed in this study abided by the Declaration of Helsinki. The institutional review board approved the study protocol, and all patients provided written informed consent.

### Surgical methods

All surgeries were accomplished by a team who was able to professionally implement hepatectomy. The surgical principles, including anatomic resection and partial resection, were followed according to the corresponding TNM classification. Proper hepatic vascular control techniques, including the selective inflow occlusion (SIO) maneuver and intermittent Pringle maneuvers (IPs), were used to reduce bleeding during liver resection. The SIO maneuver is described by the following procedure: dissecting the portal vein, proper hepatic artery, right and left hepatic arteries, and bile ducts followed by continuously blocking the hepatic artery in the tumor-bearing lobe with a bulldog clamp [21]. IPs encircling the hepatoduodenal ligament were performed with cycles of clamping and unclamping times of 15 min and 5 min, respectively. We enrolled twenty-eight patients with portal vein tumor thrombus (PVTT): 19 were type I, 6 were type II, 3 were type III, and none were type IV. The following definitions of PVTT were used: type I, tumor thrombus involving segmental branches of the portal vein or above; type II, tumor thrombus involving the right/left portal vein; and type III, tumor thrombus involving the main portal vein trunk (Cheng’s new classification system). The different therapeutic schedules of PVTT were schemed according to the corresponding type. Segmental hepatectomy was performed for type I, hemihepatectomy was performed for type II, and hepatectomy plus thrombectomy was performed for type III.

### CTC detection

The CellSearch™ system is the gold standard for capturing CTCs based on epithelial cell adhesion molecules (EpCAMs), which represent aggressive stem cell-like CTCs [22, 23].. However, a recent study showed that in the blood of a total of 14 HCC patients, the percentage of EpCAM-positive CTCs was only 8.03% [24]. The principle of the isolation by size of epithelial tumor cells (ISET) method is to isolate tumor cells by analyzing their morphology and deformability and then identify tumor cells by special staining methods. In addition to the CellSearch™ system, the ISET method is a relatively mature technology used to study CTCs. As early as 10 years ago, the ISET method was applied in breast [25] and lung cancer [26] clinical research. For HCC, the advantage of the ISET method is that it can capture EpCAM-negative tumor cells. However, it also has obvious disadvantages. In the whole study, the leucocytes and thrombus could have possibly blocked the filter pores. In our study, CTC detection was carried out in a total of 1032 blood samples (82 blood samples could not be drawn because of the patients, and the blood collection was missed). According to our statistics, due to blocked filter pores and polluted backgrounds, 8.2% (85/1032) of the blood samples failed at the isolation stage. As a result, tumor cells could not be identified. Therefore, a total of 167 CTC counts were missing from the data.

**Fig. 1:**
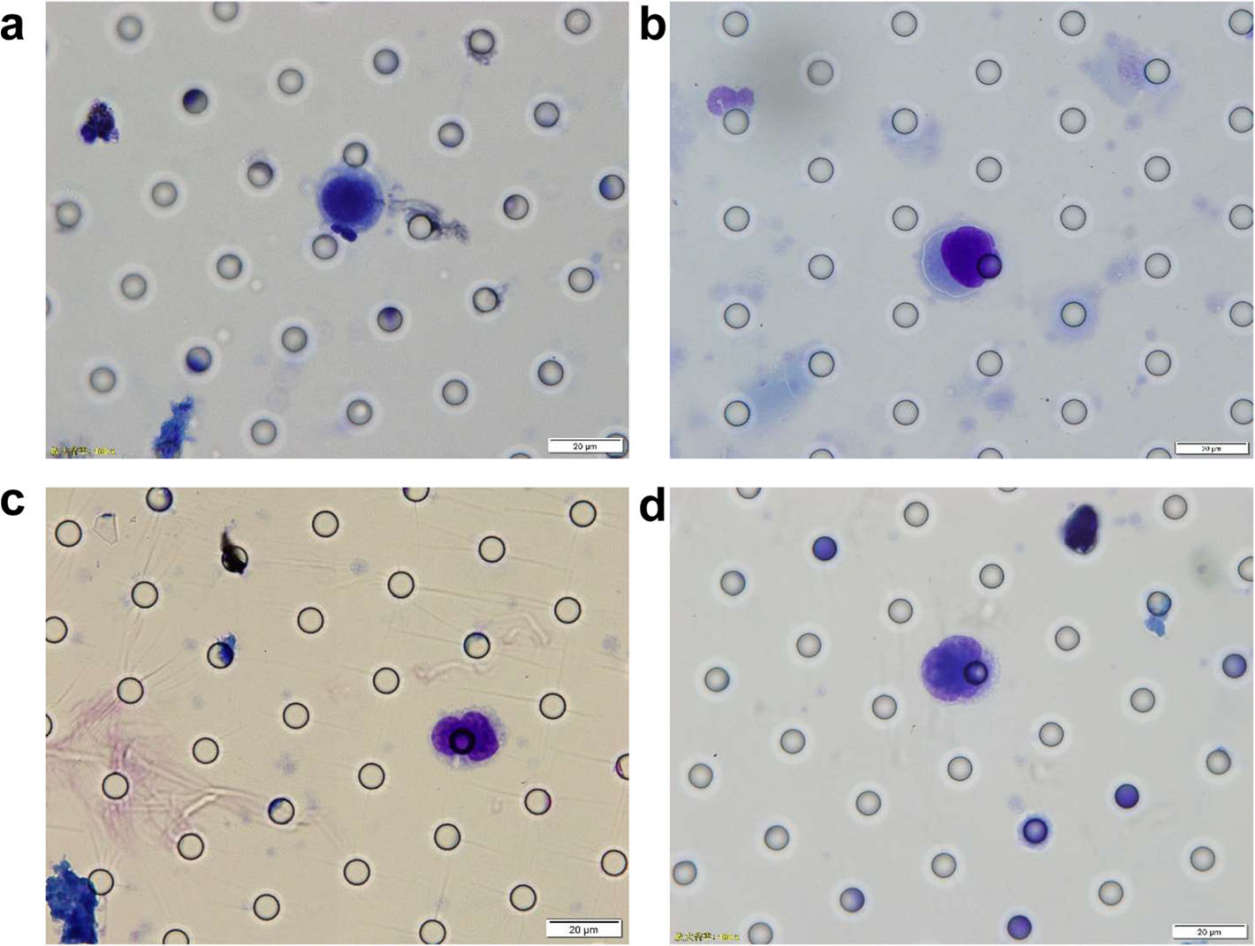
The typical microscopic images of CTCs. **a** Cell diameter larger than 15 μm and nucleus-to-cytoplasmic ratio > 0.8. **b** Abnormal karyotypes such as lobulated nuclei, dented or shriveled nuclear borders. **c** Lobulated nuclei. **d** Giant nucleoli and nucleus-to-cytoplasmic ratio > 0.8

Blood samples (5 mL) were drawn to detect CTCs by the ISET method. The time points for blood collection were before surgery (30 min before anesthesia), during surgery (30 min after tumor removal) and at 1 week, 1 month, 2 months, 3 months, 6 months, and 1 year after surgery. This method involves blood filtration and analysis by microscopy using standard histopathological/cytomorphological criteria [27, 28]. The ISET instrument filtered the blood to capture CTCs with a polycarbonate membrane with an 8 μm pore, and at least two experienced cancer cytologists finally independently analyzed and synthesized the captured CTCs. CTCs were defined with respect to the following six characteristics: a) abnormal karyotypes, such as lobulated nuclei; b) cell diameter larger than 15 μm; c) irregular, dented or shriveled nuclear borders; d) nucleus-to-cytoplasmic ratio > 0.8; e) giant nucleoli; and f) nonhomogeneous nuclear staining. Cells meeting at least four of these criteria were identified as CTCs. In addition, if giant nucleoli or abnormal karyotypes appeared and at least two other criteria were met, the cells were also identified as CTCs. Figure 1 shows typical microscopic images of CTCs.

### Follow-up and tumor recurrence

The patients were followed up every month with ultrasonography and AFP during the first 6 months after surgery and every 3 months thereafter. The patients were scheduled to have a CT scan every 6 months and an MRI scan every year. Recurrence was diagnosed by computed tomography scans, magnetic resonance imaging, digital subtraction angiography, and elevated serum alpha-fetoprotein levels. Follow-up was terminated on October 30, 2019. We defined recurrence within 1 year after surgery as an early recurrence [29]. The time to recurrence was defined as the interval between resection and the diagnosis of intrahepatic recurrence or extrahepatic metastasis. Governmental death registration and telephone follow-ups were used to determine the patient’s survival status. The mean follow-up time was 25.2 ± 6.6 months (median, 25.5 months; range, 11.6–34.3 months). Among the 137 patients, 60 were in the nonrecurrence group, 35 were in the non-early recurrence group, and 42 were in the early recurrence group. Among all 77 patients in the recurrence groups, 65 had intrahepatic recurrence only, while 12 had both intrahepatic recurrence and extrahepatic metastasis.

### Statistical analysis

The data are presented as the mean ± standard deviation (SD). Receiver operating characteristic (ROC) curve analysis was used to evaluate the predictive value of the preoperative peripheral blood CTC count for the presence of MVI. Student’s *t*-test was used for comparisons between groups where appropriate. Multivariate analysis was performed using the logistic regression model. Kaplan-Meier analysis was used to generate survival curves, and the log-rank test was used to compare patient survival between subgroups; *P < 0.05* was considered statistically significant. Statistical analyses were performed using SPSS version 19.0 for Windows (IBM).

## Results

### Patient characteristics

Table 1 details the clinical and tumor characteristics of the 137 patients with HCC. The mean patient age was 53 ± 12 years (range, 21–74 years). Of the patients, 89.8% (123/137) were male and 10.2% (14/137) were female. Hepatitis B surface antigen (HBsAg) was positive in 83.2% (114/137) of the patients, and six patients were positive for hepatitis C virus (HCV). In all, 77.4% (106/137) of the patients had liver cirrhosis, and 29.9% (41/137) of the patients had an AFP level > 400 ng/ml. The ICG-R15 (%) was 7.2 ± 4.0. The tumor diameter was 5.5 ± 3.9 cm, and 29 (21.2%) patients had multiple tumors. Portal vein thrombosis was present in 20.4% (28/137) of the patients. Tumor stage was stratified by the Barcelona Clinic Liver Cancer (BCLC) staging system. Of the patients, 56.9% (78/137) were stage 0 + A. Eight patients (5.8%) had Child-Pugh score B and received short-term liver protective therapy before surgery; the remaining patients had Child-Pugh score A.

**Table 1:** Clinical Characteristics of 137 patients

Clinical characteristics	No. of patients
Age years	Mean 53 ± 12 (21–74) Median 55
Sex	
Male	123
Female	14
ALT (U/ml)	Mean 37 ± 30 (8–96)
AST (U/ml)	Mean 30 ± 15 (14–197)
TBiL (μmol/l)	Mean 15.9 ± 9.2 (6.1–48) Median 13.4
Child-Pugh score	
A	129
B	8
HBsAg	
Positive	114
Negative	23
Liver cirrhosis	
No	31
Yes	106
ICG-R15 (%)	Mean 7.2 ± 4.0 (1–22.4) Median 6.6
AFP (ng/mL)	
≤ 400	96
> 400	41
Tumor diameter (cm)	Mean 5.5 ± 3.9 (1–15.7) Median 4.5
No. of tumor	
Single	108
Multiple	29
Portal vein tumor thrombosis	
No	109
Yes	28
BCLC stage	
0 + A	78
B + C	59

*ALT* alanine transaminase, *AST* aspartate aminotransferase, *TBiL* total bilirubin, *HBsAg* hepatitis B surface antigen, *ICG R15 min (%)* indocyanine green 15 min retention rate, *AFP* alpha fetoprotein, *BCLC* Barcelona Clinic Liver Cancer staging system

### Correlation between MVI and clinicopathological features

Table 2 shows the correlations between MVI and the clinical and tumor characteristics of the 137 patients. Univariate and multivariate analyses showed that AFP > 400 ng/ml, tumor size, and preoperative CTC count are independent risk factors for the presence of MVI.

**Table 2:** Factors for microvascular invasion on univariate and multivariate analyses

Variables	OR	Univariate Analysis		OR	Multivariate Analysis	
		HR (95% CI)	*P*		HR (95% CI)	*P*
Age	1.009	0.981 ~ 1.038	0.545		NA	NA
Sex, male versus female	0.734	0.232 ~ 2.317	0.598		NA	NA
HBsAg, positive versus negative	1.173	0.469 ~ 2.930	0.733		NA	NA
Liver cirrhosis, yes versus no	1.738	0.747 ~ 4.044	0.200		NA	NA
Child-Pugh score, B versus A	1.389	0.333 ~ 5.800	0.652		NA	NA
ALT	1.016	0.993 ~ 1.039	0.169		NA	NA
AFP (ng/ml) ≤ 400 versus > 400	14.571	5.718 ~ 37.135	< 0.001	6.702	2.149 ~ 33.354	0.002
ICG-R15 (%)	0.988	0.907 ~ 1.076	0.781		NA	NA
Tumor size	1.170	1.063 ~ 1.288	0.001	1.213	1.031 ~ 1.427	0.020
No. of tumors, multiple versus single	6.286	2.456 ~ 16.090	< 0.001	2.059	0.470 ~ 9.010	0.338
Tumor encapsulation, yes versus no	0.773	0.383 ~ 1.562	0.473		NA	NA
Edmondson stage, III-IV versus I-II	1.133	0.572 ~ 2.244	0.032	1.484	0.571 ~ 4.259	0.463
Ki67 (%)	1.027	1.011 ~ 1.044	0.003	0.994	0.968 ~ 1.021	0.664
PVTT, yes versus no	7.435	2.771 ~ 19.950	< 0.001	2.413	0.351 ~ 16.590	0.370
Satellite lesion, yes versus no	8.437	2.673 ~ 26.630	< 0.001	2.169	0.340 ~ 13.826	0.413
BCLC stage, 0 + A versus B + C	3.984	1.941 ~ 8.177	< 0.001	0.683	0.163 ~ 2.866	0.602
Preoperative CTC^5ml^	1.935	1.500 ~ 2.488	< 0.001	1.757	1.344 ~ 2.295	< 0.001

*ALT* alanine transaminase, *AFP* alpha fetoprotein, *HBsAg* hepatitis B surface antigen, *ICG R15 min (%)* indocyanine green 15 min retention rate, *PVTT* portal vein tumor thrombosis, *BCLC* Barcelona Clinic Liver Cancer staging system, *CTC* circulating tumor cell

### The clinical value of CTCs in predicting the presence of MVI

**Fig. 2:**
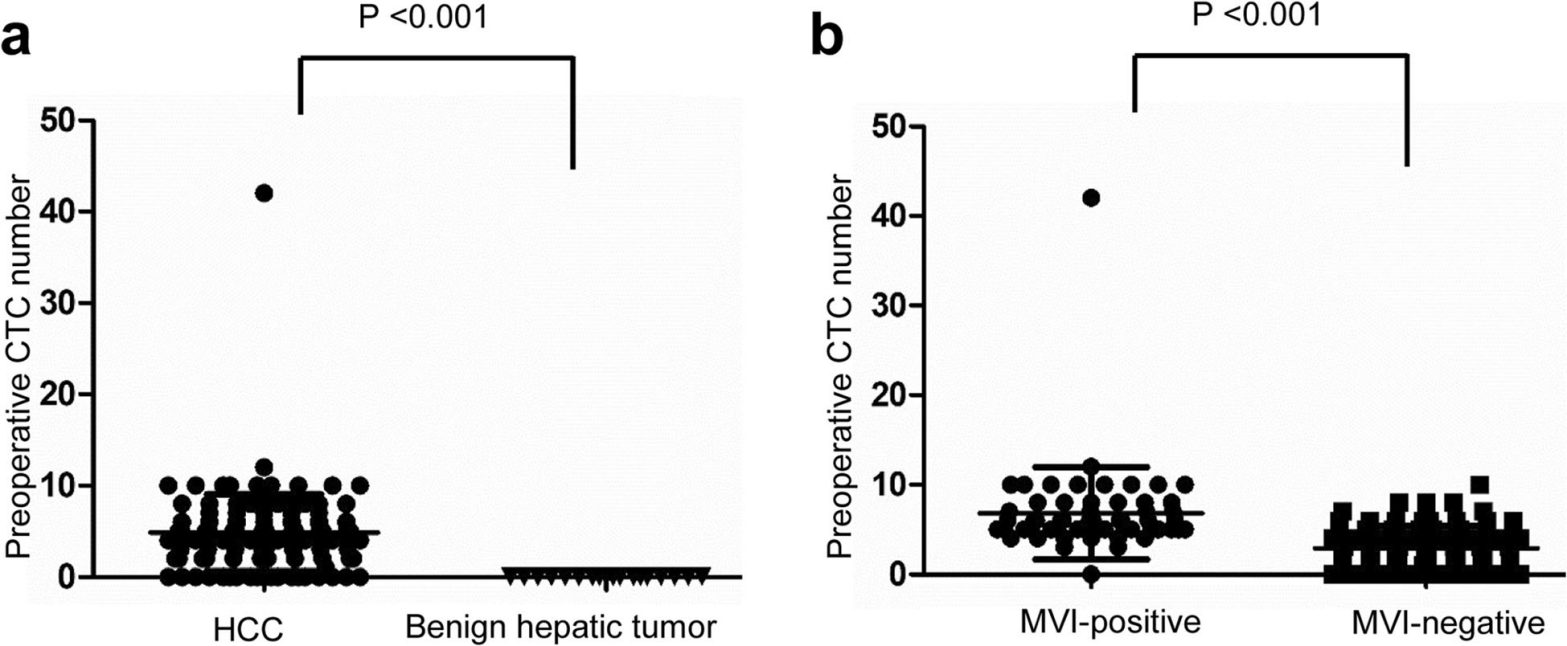
The correlation between preoperative CTC count and the presence of MVI. **a** Distribution of CTCs in patients with HCC and benign liver disease. **b** Distribution of CTCs in the MVI-positive subgroup and MVI-negative subgroup

**Fig. 3:**
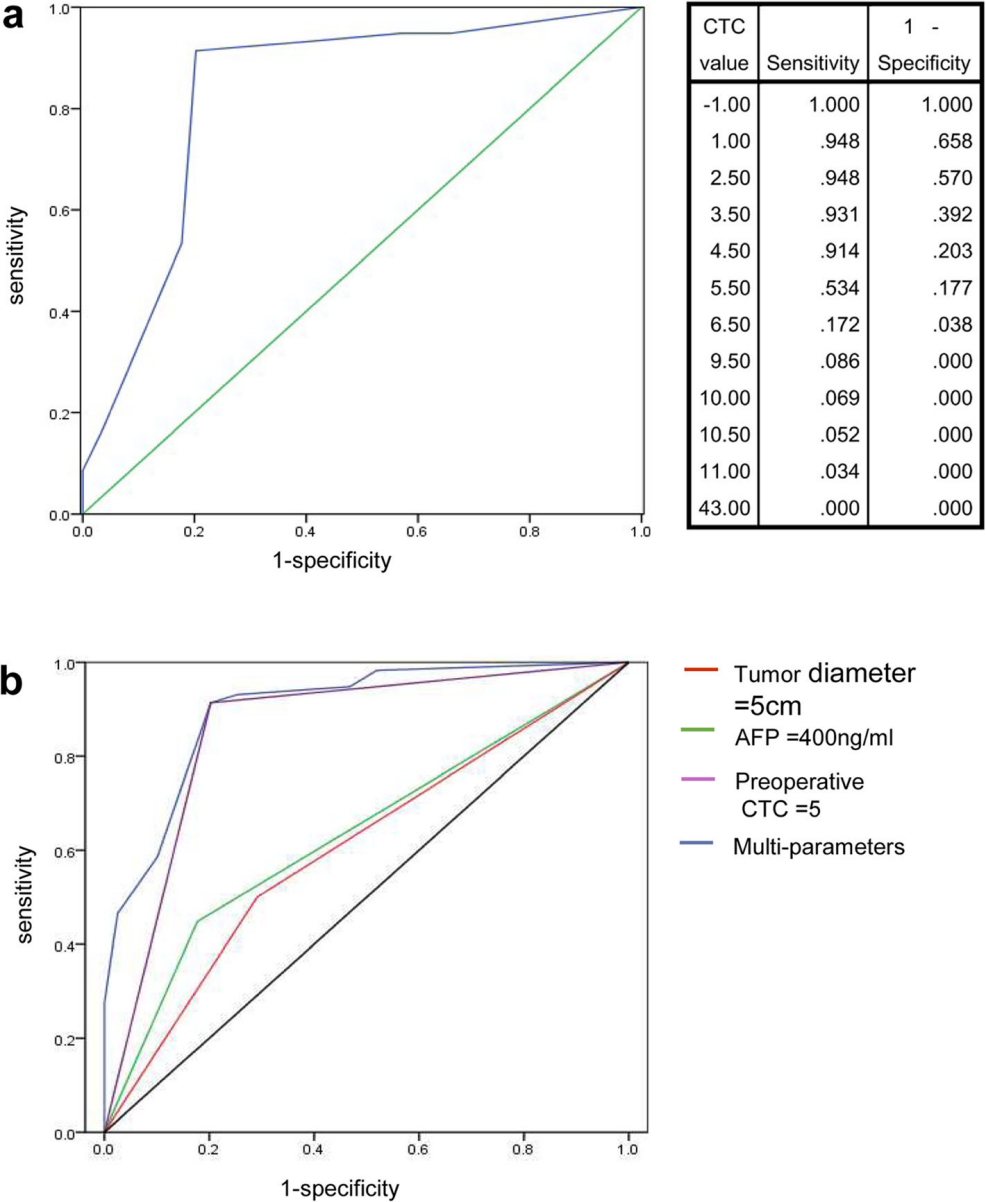
The diagnosis power of several significant parameters for predicting MVI. **a** The diagnosis power of CTC count for predicting MVI and its optimal cut-off. **b** The diagnosis power of parameters including tumor diameter ≥ 5 cm, AFP ≥ 400 ng/ml, CTC ≥ 5 and multi-parameter combination for predicting MVI

The preoperative blood sample CTC counts of patients with HCC and benign tumors are shown in Fig. 2a. A total of 18 patients with benign hepatic tumors had 0 CTCs (*P* < 0.001). A comparison of CTC counts between patients who were MVI-positive and patients who were MVI-negative is shown in Fig. 2b. The difference in the mean blood CTC levels between the MVI-positive group and the MVI-negative group was statistically significant (6.8 ± 5.1 versus 2.9 ± 2.5, *P* < 0.001). The analysis of the optimal cut-off value of CTCs is shown in Fig. 3a. The optimal cut-off value of CTCs can be calculated from the CTC value table. When CTC = 5, the sensitivity and specificity were 91.4 and 79.7%, respectively, and the Youden index reached its maximum value of 0.711. AFP, tumor diameter and preoperative CTC count were included in the model to predict the presence of MVI. The ROC curves of AFP, tumor diameter, preoperative CTC count and multiparameter combination were drawn (Fig. 3b), and the areas under the ROC curves (AUCs) of these variables were 0.636, 0.604, 0.856, and 0.900, respectively. The results indicated that a tumor diameter cut-off value of 5 and a preoperative CTC cut-off value of 5 showed the most significant power for predicting the presence of MVI. The sensitivities of AFP ≥ 400 ng/ml, tumor diameter ≥ 5 cm, preoperative CTC ≥ 5 and multiparameter combination were 44.8, 50.0, 91.4, and 91.4%, respectively, and the specificities were 82.3, 70.9, 79.7, and 79.7% (Table 3). By comparing the ROC curve features and AUCs, the results showed that compared with AFP, tumor diameter and CTC, the multiparameter combination had the most significant power for predicting the presence of MVI.

**Table 3:** The parameters for predicting the presence of MVI

	Sensitivity	Specificity	AUC	Cut-off value
AFP	44.8%	82.3%	0.636	400 ng/ml
Tumor diameter	50.0%	70.9%	0.604	5 cm
Preoperative CTC	91.4%	79.7%	0.856	5
Multi-parameter	91.4%	79.7%	0.900	–

*AFP* alpha fetoprotein, *CTC* circulating tumor cell

### The impact of the presence of MVI on the prognosis of HCC

**Fig. 4:**
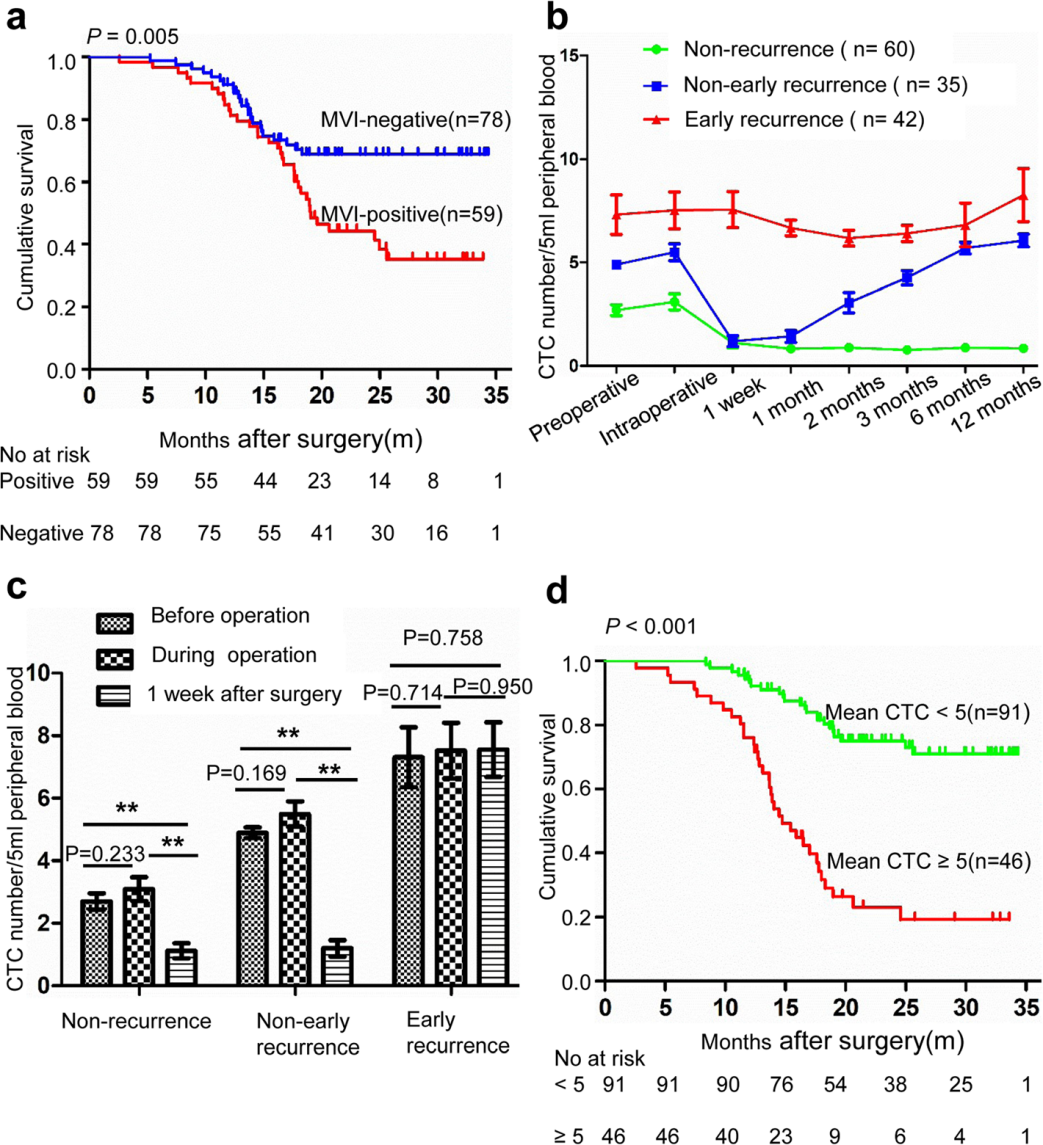
CTC number change and prognosis. **a** Kaplan-Meier analysis for time to recurrence in patients with HCC who were MVI-positive and MVI-negative. **b** The dynamic change of three groups (no recurrence [*n* = 60], non-early recurrence [*n* = 35] and early recurrence [*n* = 42]) with regard to CTC number at all time points. **c** The CTC number change in the three groups before, during and 1 week after surgery (** = *P* < 0.05). **d** Kaplan-Meier analysis for time to recurrence in patients with HCC with mean CTC ≥ 5 and CTC < 5

Of the 137 patients, 59 were MVI positive. The MVI-positive group showed significantly shorter OS than the MVI-negative group (median OS 19.2 months, 95% CI 17.8–22.1 months versus not reached, *P* = 0.005) (Fig. 4a).

### Comparison the change of CTC count caused by surgery in the subgroups

All patients were divided into three groups: no recurrence (*n* = 60), non-early recurrence (*n* = 35) and early recurrence (*n* = 42) groups. Figure 4b shows the CTC counts at each time point for the three groups. Figure 4c shows that the intraoperative CTC counts did not increase across the three groups compared to the preoperative values (2.7 versus 3.1, *P* = 0.233; 4.9 versus 5.5, *P* = 0.169; 7.3 versus 7.5, *P* = 0.714). The number of CTCs in the nonrecurrent group and the non-early recurrent group decreased significantly 1 week after surgery compared with that in the intraoperative group (1.1 versus 3.1, *P* < 0.001; 1.2 versus 5.5, *P* < 0.001), although there was no significant difference in the early recurrence group (7.6 versus 7.5, *P* = 0.950). Using a mean CTC count of 5 as the cut-off value, all patients were divided into two groups. The survival curve showed that the survival time of the CTC count ≥5 group was significantly shorter than that of the CTC count < 5 group (14.7 months, 95% CI 12.1–15.6 months versus not reached, *P* < 0.001) (Fig. 4d). The proportion of early recurrence cases in the CTC count ≥5 group was also higher than that in the CTC count < 5 group (84.8% versus 3.3%, *P* < 0.001). When the mean value of CTCs is greater than 5 and remains greater than 5 after surgery, it strongly indicates a risk of early postoperative recurrence. Extrahepatic metastasis occurred in 12 of the 137 patients. We defined the difference between the CTC count 1 week after surgery and the intraoperative CTC count as ΔCTC. We found that the number of CTCs in the non-extrahepatic metastasis group decreased 1 week after surgery, but the number of CTCs in the extrahepatic metastasis group increased abnormally 1 week after surgery (− 2.4 ± 2.5 versus 2.3 ± 2.8, *P* < 0.001).

## Discussion

An absolutely safe surgical margin is a prerequisite for the complete removal of residual microtumor thrombosis. Anatomic resection independently improves long-term survival in patients with tumor diameters ranging from 2 cm to 5 cm, which is probably due to the elimination of MVI in the resected domain [30]. However, the excessive removal of non-neoplastic liver parenchyma can lead to liver dysfunction and the comorbidities of ascites, jaundice and hypoalbuminemia. Therefore, it is important to identify the subpopulation of patients with HCC at high risk of MVI preoperatively to instruct precise hepatectomy. In the present study, we found that patients with preoperative CTC counts ≥5 were more likely to have MVI. Compared to AFP, tumor diameter, and preoperative CTC count, the multiparameter combination was the most significant predictor of MVI and helped to guide the options for surgical methods.

It is controversial whether surgical manipulation can drive CTCs into the blood, resulting in the dissemination of tumor cells. Previous studies showed that the CTC value tended to decrease postoperatively until it was maintained at normal levels [19, 31]. Other studies have demonstrated that an increase in postoperative CTCs indicates a poor prognosis [32, 33]. We found that the intraoperative CTC level of the patients in the three groups did not increase compared to the preoperative CTC level. The results showed that intraoperative manipulation by the surgeon does not drive CTCs into the bloodstream, resulting in the dissemination of tumor cells. In the nonrecurrence and non-early recurrence subgroups, we found that the level of CTCs at 1 week after surgery decreased sharply compared to intraoperative levels and remained at a low level until recurrence. The results suggested that the removal of the tumor was one of the reasons for the decrease in CTCs 1 week after surgery and that recurrence will lead to a subsequent increase. However, in the early recurrence subgroup, the CTC levels did not decrease 1 week after surgery and remained at a consistently high level. In addition, in the extrahepatic metastasis group, CTC increased abnormally 1 week after surgery. However, in the early recurrence subgroup, the CTC levels did not decrease 1 week after surgery and remained at a consistently high level. In addition, in the extrahepatic metastasis group, CTCs increased abnormally 1 week after surgery. In our data, of the 12 patients with extrahepatic metastasis, nine experienced extrahepatic metastasis within 1 year after surgery. By analyzing the dynamic changes in the number of CTCs, we found that the average CTC level of patients with early recurrence was higher than that of patients in the other subgroups; patients with an average CTC level greater than 5 had a significantly higher risk of early recurrence and a significantly shorter survival time than those in the other subgroups.

The limitations of this study are the small sample size, short follow-up time, and single study center. A multicenter, large sample, randomized clinical trial should be designed to illustrate the prognostic significance of CTCs in HCC.

## Conclusion

The preoperative CTC counts in the peripheral blood of patients with HCC are closely correlated with MVI. The intraoperative manipulation of the lesion by the surgeon does not increase the number of CTCs in peripheral blood. Surgical removal of the tumor decreases the number of CTCs. The persistence of CTCs at a high level (≥ 5) after surgery suggests a risk of early recurrence.

## Data Availability

All data generated or analyzed during this study are available from the corresponding author.

## Abbreviations

ALT: Alanine transaminase
AST: Aspartate aminotransferase
AFP: Alpha fetoprotein
BCLC: Barcelona Clinic Liver Cancer staging system
CTC: Circulating tumor cell
CT: Computerized tomography
EMT: Epithelial and mesenchymal transition
EpCAM: Epithelial cell adhesion molecules
HCC: Hepatocellular carcinoma
HBsAg: Hepatitis B surface antigen
ISET: Isolation by size of epithelial tumor cells
ICG-R15: Indocyanine green 15 min retention rate
IPs: Intermittent Pringle maneuvers
MVI: Microvascular invasion
MRI: Magnetic resonance imaging
OS: Overall survival
PIVKA-II: Prothrombin induced by vitamin K absence-II
PVTT: Portal vein tumor thrombosis
ROC: Receiver operating characteristic curve
SIO: Selective inflow occlusion maneuver
TBiL: Total bilirubin
